# Phytochemical Composition and Antioxidant Activities of Two Different Color Chrysanthemum Flower Teas

**DOI:** 10.3390/molecules24020329

**Published:** 2019-01-17

**Authors:** Ah-Reum Han, Bomi Nam, Bo-Ram Kim, Ki-Chang Lee, Beom-Seok Song, Sang Hoon Kim, Jin-Baek Kim, Chang Hyun Jin

**Affiliations:** Advanced Radiation Technology Institute, Korea Atomic Energy Research Institute, Jeongeup-si, Jeollabuk-do 56212, Korea; bomi1201@kaeri.re.kr (B.N.); boram1606@kaeri.re.kr (B.-R.K.); kichang21@kaeri.re.kr (K.-C.L.); sbs0110@kaeri.re.kr (B.-S.S.); shkim80@kaeri.re.kr (S.H.K.); jbkim74@kaeri.re.kr (J.-B.K.); chjin@kaeri.re.kr (C.H.J.)

**Keywords:** *Chrysanthemum* tea, HPLC-DAD-ESIMS, HS-SPME-GC-MS, anthocyanin, flavonoid, phenolic acid, volatile, antioxidant

## Abstract

*Chrysanthemum morifolium* Ramat is a perennial flowering plant widely cultivated for use in a tea infusion and as a popular beverage. To identify and evaluate the tea infusion made with a γ-irradiated mutant chrysanthemum cultivar with dark purple petals (cv. ARTI-Dark Chocolate), its phytochemical composition and antioxidant activity were tested and compared with those of the commercially available chrysanthemum cultivar with yellow petals (cv. Gamguk) by HPLC-DAD-ESIMS, as well as DPPH and ABTS radical scavenging assays. The purple chrysanthemum tea contained anthocyanins and linarin, which were not detected in the yellow chrysanthemum tea and the content of chlorogenic acid, acacetin-7-*O*-β-glucoside, and luteolin was higher compared with the yellow chrysanthemum tea. In contrast, the yellow chrysanthemum tea had higher luteolin-7-*O*-β-glucoside, 3,5-dicaffeoylquinic acid, apigenin-7-*O*-β-glucoside, and apigenin contents in comparison with the purple chrysanthemum tea. In addition, the content and antioxidant activity of the two chrysanthemum teas were investigated according to different water temperatures and infusing time. The yellow chrysanthemum tea did not show any significant differences according to infusing time and temperature, while the purple chrysanthemum tea was more influenced by the infusing time than water temperature, showing the highest total compound content in the infusing condition of 100 °C and 4 min. Moreover, the floral scent volatiles of the two chrysanthemum tea sources were analyzed using HS-SPME-GC-MS. In the DPPH radical scavenging assay, the purple chrysanthemum tea broadly showed greater antioxidant activity than did the yellow chrysanthemum tea, corresponding to the high content of anthocyanins known as the powerful antioxidant. Further, both chrysanthemum flower teas exhibited strong ABTS radical scavenging effects ranging from 76% to 61% under all infusing conditions. Therefore, the purple chrysanthemum cultivar, ARTI-Dark Chocolate, is worthy of breeding as a new tea cultivar.

## 1. Introduction

In Asia, chrysanthemum flower tea prepared from the flowers of *Chrysanthemum morifolium* Ramat. (Asteraceae) is a popular beverage due to its desirable taste and aroma. Further, it has reported health benefits as chrysanthemum flowers possess anti-inflammatory, anti-pyretic, sedative, anti-arthritic, and anti-hypertensive effects [1]. Chrysanthemum flowers have also been demonstrated to produce various types of flavonoids [2,3,4,5], phenolic acids [2,6,7,8], and lignans [9], which have exhibited diverse biological activities such as antioxidant [3,6], anti-inflammatory [4], antitumor [5], neuroprotective [7,9], and anti-allergic activities [10]. The properties and concentrations of such constituents are responsible for the functional characteristics of chrysanthemum and are important from a commercial point of view. 

Although chrysanthemum flowers with yellow petals (e.g., Gamguk and Sanguk) are usually used for tea material and make the greatest contribution of an antioxidant and a coolant to the Korean herb tea market according to their frequency of consumption, there has been little research on the development of chrysanthemum tea using various colors of chrysanthemum flowers. Therefore, evaluation of new tea plants using active compound profiles that affect tea quality is an important method for new tea plant breeding and commercialization. 

**Figure 1:**
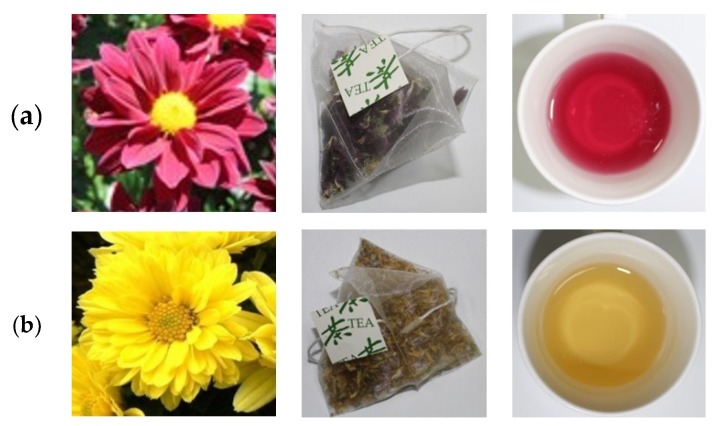
Photos of chrysanthemum flowers in different colors. (**a**) Dark purple chrysanthemum cultivar (ARTI-Dark Chocolate, ADC), its manufactured tea bag, and its infusion; (**b**) Common yellow chrysanthemum cultivar (Gamguk, GG), its manufactured tea bag, and its infusion.

Recent γ-irradiated mutation breeding studies on chrysanthemum cultivars performed at the Advanced Radiation Technology Institute (Republic of Korea) resulted in the development of a chrysanthemum flower with dark purple petals (cv. ARTI-Dark Chocolate), which obtained a plant variety protection right (registration No. 4996) on April 15, 2014, through examination by the Korea Seed and Variety Service. In the present study, to identify and evaluate the two different color varieties, ARTI-Dark Chocolate (ADC) and Gamguk (GG), tea infusion samples were prepared according to different temperatures and times (Figure 1). The content and composition of bioactive compounds in the samples were analyzed using high performance liquid chromatography-diode array detector-electrospray ionization mass spectrometry (HPLC-DAD-ESIMS) and their antioxidant properties were tested using the 1,1-diphenyl-2-picryl-hydrazyl (DPPH) and 2,2-azino-bis-3-ethylbenzothiazoline-6-sulfonic acid (ABTS) radical scavenging assays. In addition, we performed headspace solid-phase microextraction coupled with gas chromatography-mass spectrometry (HS-SPME-GC-MS) to analyze the floral scent volatiles from the two different color varieties using dried flower materials in ADC and GG tea bags. The aim of our investigation was not only to identify the advantages of this new chrysanthemum cultivar as a tea ingredient, but also to provide a new viewpoint for its further application. 

## 2. Results and Discussion

### 2.1. Identification of Phytochemical Components in Two Different Color Chrysanthemum Flower Teas

#### 2.1.1. Flavonoids and Phenolic Acids

**Figure 2:**
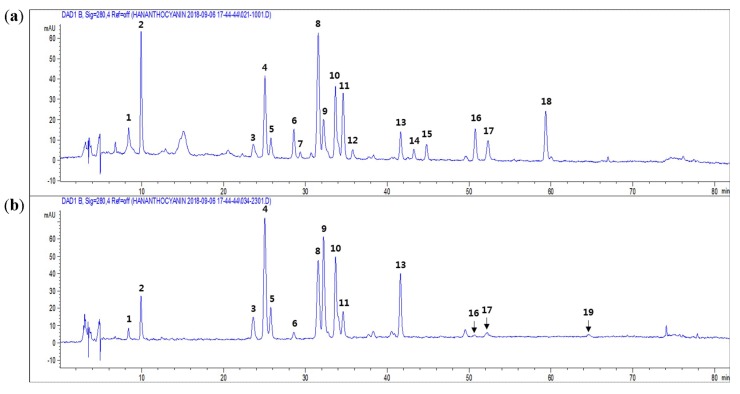
HPLC chromatograms of (**a**) the dark purple chrysanthemum cultivar (ARTI-Dark Chocolate) infusion and (**b**) the yellow chrysanthemum cultivar (Gamguk) infusion detected at 280 nm. See Table 1 for the peak numbers and Materials and Methods for the HPLC-DAD-ESIMS conditions.

To characterize flavonoids and phenolic acids, the freeze-dried concentrates of infusions from two different color chrysanthemum flowers were subjected to HPLC-DAD-ESIMS analysis. Nineteen peaks were separated in the HPLC chromatograms (Figure 2). These peaks were characterized by typical UV absorptions obtained from the diode array detector, with the maximum absorptions at 250–270 and 330–350 nm for flavonoids and at 220 and 325 nm for phenolic acids (Table 1). Compounds corresponding to nine of the peaks were identified as chlorogenic acid (peak 2), luteolin-7-*O*-β-glucoside (peak 4), 3,5-dicaffeoylquinic acid (peak 8), apigenin-7-*O*-β-glucoside (peak 9), linarin (peak 17), acacetin-7-*O*-β-glucoside (peak 18), luteolin (peak 19), apigenin (peak 20), and acacetin (peak 22), by comparison of the retention time, UV spectra, and positive molecular ions with those of the compounds isolated or purchased in our previous study [11] (Table 1). The remaining peaks were unknown compounds and were tentatively identified by molecular ion and fragment ion assignment together with the characteristic UV absorption using published reports [2,8,9] (Table 1). Peaks 1, 3, 5, 10, 12, 13, 14, and 19 showed UV absorptions of flavonoids at 265–275 and 325–335 nm. Further, MS chromatograms exhibited a positive molecular ion at *m*/*z* 449 [M + H]^+^ for peak 1, a positive molecular ion at *m*/*z* 465 [M + H]^+^ for peak 3, a positive molecular ion at *m*/*z* 463 [M + H]^+^ for peak 5, a positive molecular ion at *m*/*z* 535 [M + H]^+^ for peak 10, a positive molecular ion at *m*/*z* 477 [M + H]^+^ for peak 12, a positive molecular ion at *m*/*z* 519 [M + H]^+^ for peak 13, a positive molecular ion at *m*/*z* 549 [M + H]^+^ for peak 14, and a positive molecular ion at *m*/*z* 533 [M + H]^+^ for peak 18, suggesting that they were luteolin-glucoside (peak 1), quercetin-glucoside (peak 3), luteolin-glucuronide (peak 5), luteolin-malonylglucoside (peak 10), diosmetin-glucuronide (peak 12), apigenin-malonylglucoside (peak 13), diosmetin-malonyglucoside (peak 14), and acacetin-malonylglucoside (peak 18). Peaks 6 and 11 had UV absorptions at 220–225 and 325 nm and a positive molecular ion at *m*/*z* 517 [M + H]^+^ and a fragment ion at *m*/*z* 499 (loss of H_2_O group 18 mass unit) and 163 (loss of chlorogenic acid group 354 mass unit), suggesting dicaffeoylquinic acid (peaks 6 and 10). However, the substituted positions and configuration of dicaffeoyl groups could not be determined by UV and MS spectral data analysis. UV maxima absorptions at these wavelengths of such flavonoids and phenolic acids were not observed in peak 7 but its mass spectra showed positive molecular ions at *m*/*z* 551 [M + H]^+^ and a fragment ion at *m*/*z* 295. A significant difference in the HPLC-DAD profiles between the two different color chrysanthemum flower teas is that peaks 7, 12, 14, and 15 were clearly detected in the purple chrysanthemum flower tea, but not in the yellow chrysanthemum flower tea. In contrast, the LC chromatogram of the yellow flower tea showed a clear detection of apigenin (peak 19), which was not detected in the purple flower tea. 

#### 2.1.2. Anthocyanins

**Figure 3:**
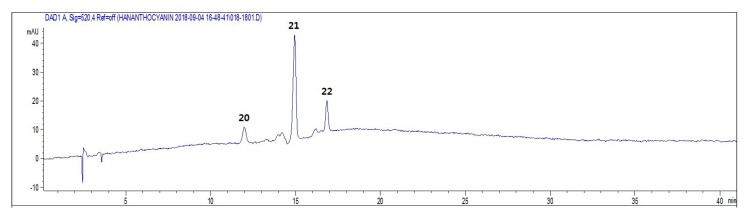
HPLC chromatograms of the dark purple chrysanthemum cultivar (ARTI-Dark Chocolate) infusion detected at 520 nm. See Table 2 for the peak numbers and Materials and Methods for the HPLC-DAD-ESIMS conditions.

Identification of anthocyanins was made based on comparison of retention times and UV/Vis and mass spectra for either authentic standards (Figure 3 and Table 2). The anthocyanin peaks were characterized by typical UV absorptions at 520 nm and corresponded to cyanidin-3-*O*-glucoside (peak 20), cyanidin 3-*O*-(6″-malonylglucoside) (peak 21), and cyanidin (peak 22) by retention time. Their MS chromatograms also exhibited a positive molecular ion at *m*/*z* 449 [M + H]^+^ for peak 20, *m*/*z* 535 [M + H]^+^ for peak 21, and *m*/*z* 287 [M + H]^+^ for peak 22. These cyanidin 3-*O*-glucoside, cyanidin 3-*O*-(6″-malonylglucoside), and cyanidin peaks were clearly detected in the purple chrysanthemum flower tea LC chromatograms, but not in those of the yellow chrysanthemum flower tea.

### 2.2. Quantitative Analysis of Phytochemical Components in Two Different Color Chrysanthemum Flower Teas

**Figure 4:**
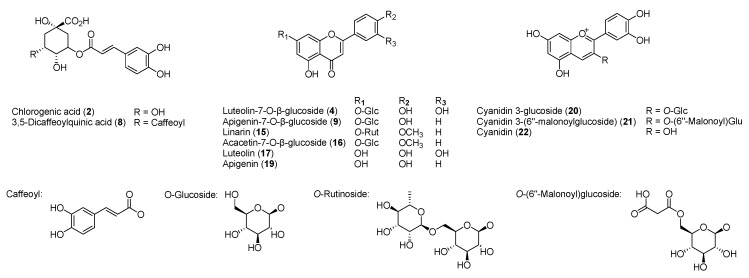
Structures of 11 standard compounds identified in two different color chrysanthemum flowers.

Using the HPLC-DAD method, quantification of the two phenolic acids, six flavonoids, and three anthocyanins that were identified from infusions of two different color chrysanthemum flowers through comparison with standards (Figure 4) was performed. 

The compound number was given the same as the peak number shown in Table 1 and Table 2 and Figure 2 and Figure 3. The detection wavelength for quantification of these compounds was chosen as 280 nm for the phenolic acids and flavonoids and 520 nm for the anthocyanins by comparison of the sample solution chromatograms at different wavelengths. Good linear calibration curves were obtained with the 11 tested standard compounds (Table 3).

Owing to the unsatisfactory separation of the 1,4-, 1,5-, and 3,5-dicaffeoylquinic acids, their total content was calculated by calibration of 3,5-dicaffeoylquinic acid. The contents of 11 compounds in infusions of two different color chrysanthemum flowers with different infusion conditions were summarized in Table 4. The ADC flower infusions had higher contents of chlorogenic acid (**1**), linarin (**5**), acacetin-7-*O*-β-glucoside (**6**), and luteolin (**7**) than the GG flower infusions, while the contents of luteolin-7-*O*-β-glucoside (**2**), 3,5-dicaffeoylquinic acid (**3**), apigenin-7-*O*-β-glucoside (**4**), and apigenin (**8**) were higher in the GG flower infusions. Compared with the total amount of phenolic acids and flavonoids, the ADC flower infusions had their contents ranging from 16 to 39 mg/g, while the GG flower infusions contained larger amounts ranging from 32 to 41 mg/g. Anthocyanins, cyanidin-3-*O*-glucoside (**9**), cyanidin 3-*O*-(6″-malonylglucoside) (**10**), and cyanidin (**11**), which were not contained in the GG flower infusions were present in the ADC flower infusions at a total content of 2–12 mg/g. Thus, it is considered that anthocyanins were extracted from the ADC flowers instead of phenolic acids and flavonoids relatively, inferred from the color of the ADC flowers.

To identify for the optimum infusing condition to extract the bioactive compounds, two chrysanthemum flowers were infused at different temperatures and times. The comparison of the amount of extracted compounds according to the infusing temperature-time relation is summarized in Table 4. In the ADC flower tea infusions, the content of compounds, apart from **4**, **21**, and **22,** increased slightly when the infusing time increased from 2 min to 4 min at 75 °C, and the contents of **4**, **21**, and **22** increased about 1.2, 3, and 2 times, respectively. At 100 °C, all compound contents almost doubled as the infusing time increased. When the infusing time was equal to 2 min and the infusing temperature increased from 75 °C to 100 °C, the contents of all compounds tended to be almost the same or slightly decreased. When infused for 4 min, the contents of all compounds increased almost twice or more than two times as the infusing temperature increased. Unlike the ADC flower tea, the GG flower tea showed no significant variation in the content of compounds according to the infusing temperature and time, although the total phenolic acid and flavonoid contents were higher than those of the ADC flower tea. However, when infused at 100 °C for 4 min, the ADC flower tea contained a higher amount of total phenolic acids compared with the GG flower tea. Comprehensively, when infused at 75 °C for 2 min and 4 min and at 100 °C for 2 min, the active compound contents extracted from the GG flower tea were higher than those of the ADC flower tea including the total anthocyanin content detected only in the ADC flower tea. When infused at 100 °C for 4 min, large amounts of anthocyanins and phenolic compounds were extracted from the ADC flower tea, thus its total compound content was higher than that of the GG flower tea. The GG flower tea did not show any significant differences according to infusing time and temperature, while the ADC flower tea was influenced by the infusing time rather than water temperature, showing the highest total compound content in the infusing condition of 100 °C and 4 min.

### 2.3. Antioxidant Activities of Two Different Color Chrysanthemum Flower Teas

The two different color chrysanthemum flower teas were evaluated for their antioxidant activity using the ATBS and DPPH radical scavenging assays. As shown in Table 5, the purple chrysanthemum cultivar ADC flower tea showed higher DPPH radical scavenging activity than did the yellow chrysanthemum cultivar GG flower tea and its infusion at 100 °C for 4 min had greater activity than that of a positive control, ascorbic acid. In the ATBS radical scavenging test results, the two chrysanthemum flower teas exhibited strong inhibitory effects ranging from 61% to 76% under all infusing conditions.

### 2.4. Identification of Floral Scent in Two Different Color Chrysanthemum Flower Tea Bags

**Figure 5:**
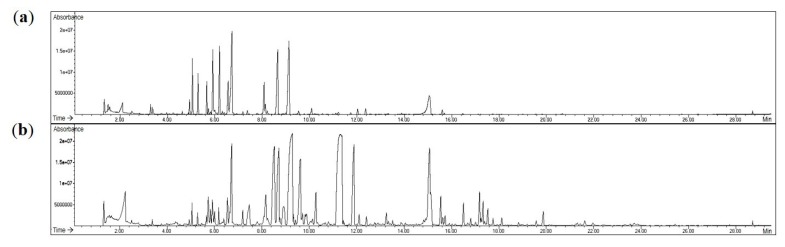
GC chromatograms of (**a**) the dark purple chrysanthemum cultivar (ARTI-Dark Chocolate) tea material and (**b**) the yellow chrysanthemum cultivar (Gamguk) tea material. See Table 1 for the peak identification and Materials and Methods for the HS-SPME-GC-MS conditions.

Floral scent volatiles in the dried flower materials of ADC and GG tea bags were extracted by headspace solid-phase microextraction (HS-SPME) and identified by gas chromatography-mass spectrometry (GC-MS) (Figure 5). HS-SPME allows for simple and rapid extraction of volatile compounds and a high reproducibility under the same conditions. The volatile compounds were affected by sample weight, different temperatures, type of extraction fibers, extraction time, and balance periods. By referring to several previous studies on the analysis of the volatile compounds in chrysanthemum flowers [12,13,14], we performed HS-SPME-GC-MS experiments by modifying their conditions (see Section 3.5). The relative contents of volatile compounds were detected by % of total area (Table 6). A total of 29 compounds were identified from ADC tea material, which accounted for 82.74% of total volatile compounds. The components with the highest contents were as follows: eucalyptol (16.25%), (+)-2-bornanone (14.33%), chrysanthenone (11.34%), β-myrcene (7.44%), and α-phellandrene (6.65%). In the GC-MS analysis of GG tea material, 49 compounds were identified, which accounted for 74.93% of total latile compounds. The components with the highest contents were as follows: Trans-verbenyl acetate (16.24%), (+)-2-bornanone (12.28%), β-elemene (7.02%), pseudo-cyclocitral (6.22%), and chrysanthnone (5.49%). The proposed HS-SPME-GC-MS can be considered at least as an alternative scenery quality assessment tool for the aroma evaluation of chrysanthemum tea materials.

## 3. Materials and Methods 

### 3.1. General Procedures

Analytical HPLC-DAD-ESIMS was carried out on an Agilent 1200 series system and an Agilent 6120 quadrupole MS system (Agilent Technologies Co., Santa Clara, CA, USA) equipped with a YMC-Triart C18 column (5 μm, 250 mm × 4.6 mm, YMC Co., Tokoy, Japan). A [^60^Co] γ-irradiator (150 TBq capacity; ACEL, MDS Nordion, ON, Canada) was used for γ-irradiation. Standards of anthocyanins (cyanidin chloride and cyanidin 3-*O*-glucoside chloride) were purchased from Sigma (St Louis, MO, USA). Cyanidin 3-*O*-(6″-malonylglucoside) was obtained from Cfm Oskar Tropitzsch GmbH (Marktredwitz, Germany). Chlorogenic acid and 1,4-dicaffeoylquinic acid were purchased from Wuhan ChemFaces Biochemical Co., Ltd. (Wuhan, Hubei, China). 1,5- and 3,5- dicaffeoylquinic acid were obtained from Chengdu Biopurify Phytochemicals Ltd. (Chengdu, China). Flavonoids (apigenin, acacetin, luteolin, apigenin-7-*O*-glucoside, acacetin-7-*O*-glucoside, luteolin-7-*O*-glucoside, linarin) were isolated from the flowers of *C. morifolium* cv. ARTI-Dark Chocolate in our previous study [11]. 2,2-Diphenyl-1-picrylhydrazyl (DPPH), 2,20-azino-bis(3-ethylbenzthiazoline-6-sulphonic acid) (ABTS), and ascorbic acid were obtained from Sigma Chemical Co. (St. Louis, MO, USA). Dulbecco’s Modified Eagle’s Medium (DMEM) and fetal bovine serum (FBS) were purchased from Hyclone (Logan, UT, USA). All other chemicals and solvents used in this study were of analytical grade. 

### 3.2. Plant Materials

A purple chrysanthemum flower with single dark purple petals (cv. ARTI-Dark Chocolate; ADC) was developed by γ-irradiation (50 Gy) from a ^60^Co source on stem cuttings of the Noble Wine cultivar with single white petals with purple stripes, followed by selection of petal-color variants and examination of stable phenotype inheritance for 4 years (2009–2012) at the Advanced Radiation Technology Institute, an affiliated institute of the Korea Atomic Energy Research Institute. Fully-open flowers, including the calyxes, were harvested on 25 August 2017. Commercially-marketed yellow chrysanthemum flower tea bags in Korea are produced using the Gamguk cultivar (GG) with all-doubled or single yellow petals. And it is the progenies of natural hybrids of *C. morifolium*. The two different chrysanthemum flowers were dried using a cold air drying method (air conditioned at a temperature of 15 °C and relative humidity of 20%). Dried chrysanthemum flowers of ADC and GG weighing approximately 1 g each were randomly selected and then made into tea bag pouches.

### 3.3. Preparation of Sample and Standard Solutions

Each chrysanthemum flower tea bag (1 g) was infused in 100 mL of distilled water at different temperatures and times. Distilled water was heated at 75 (the temperature of the water purifier) and 100 °C and each tea sample was brewed for 2 and 4 min. Each tea sample was prepared in triplicate. The infusions were evaporated in vacuo and freeze-died to afford dryness to each (ADC1 (75 °C, 2 min), 105.2 mg, *w*/*w* 10.52%; ADC2 (75 °C, 2 min), 100.6 mg, *w*/*w* 10.06%; ADC3 (75 °C, 2 min), 123.3 mg, *w*/*w* 12.33%; ADC1 (75 °C, 4 min), 134.6 mg, *w*/*w* 13.46%; ADC2 (75 °C, 4 min), 111.2 mg, *w*/*w* 11.12%; ADC3 (75 °C, 4 min), 124.1 mg, *w*/*w* 12.41%; ADC1 (100 °C, 2 min), 89.0 mg, *w*/*w* 8.9%; ADC2 (100 °C, 2 min), 108.9 mg, *w*/*w* 10.89%; ADC3 (100 °C, 2 min), 110.0 mg, *w*/*w* 11%; ADC1 (100 °C, 4 min), 191.1 mg, *w*/*w* 19.11%; ADC2 (100 °C, 4 min), 175.5 mg, *w*/*w* 17.55%; ADC3 (100 °C, 4 min), 140.8 mg, *w*/*w* 14.08%; GG1 (75 °C, 2 min), 187.5 mg, *w*/*w* 18.75%; GG2 (75 °C, 2 min), 270.5 mg, *w*/*w* 27.05%; GG3 (75 °C, 2 min), 148.7 mg, *w*/*w* 14.87%; GG1 (75 °C, 4 min), 205.6 mg, *w*/*w* 20.56%; GG2 (75 °C, 4 min), 217.3 mg, *w*/*w* 21.73%; GG3 (75 °C, 4 min), 208.7 mg, *w*/*w* 20.87%; GG1 (100 °C, 2 min), 177.0 mg, *w*/*w* 17.7%; GG2 (100 °C, 2 min), 166.3 mg, *w*/*w* 16.63%; GG3 (100 °C, 2 min), 164.8 mg, *w*/*w* 16.48%; GG1 (100 °C, 4 min), 180.5 mg, *w*/*w* 18.05%; GG2 (100 °C, 4 min), 210.5 mg, *w*/*w* 21.05%; GG3 (100 °C, 4 min), 222.6 mg, *w*/*w* 22.26%). All samples were weighed accurately and dissolved in 50% MeOH at 2 mg/mL. The sample solution was filtered through a syringe filter (0.45 μm) for LC-MS analysis. The standards were weighed accurately and dissolved in MeOH at 1.0 mg/mL. The stock solutions were diluted to yield a series of standard solutions at different concentrations for quantitative analysis.

### 3.4. Analysis of Chemical Composition Using HPLC-DAD-ESIMS

Analysis of the chemical composition of the samples was conducted using the Agilent 1200 series LC system coupled on-line with an Agilent 6120 quadrupole single mass spectrometer detector. Data acquisition and processing were performed using the ChemStation software, with an YMC-Triart C18 column (5 μm, 250 mm × 4.6 mm, YMC Co.). Binary gradient elution with 0.1% formic acid in water (*v*/*v*, solvent A) and 0.1% formic acid in acetonitrile (*v*/*v*, solvent B) was performed as follows: 0–60 min, 15–35% B; 60–70 min, 35–60% B; 70–71 min, 60–95% B; 71–80 min, 95% B; 80–81 min, 95–15% B; 81–90 min, 15% B. The total flow rate was maintained at 0.8 mL/min and the injection volume was 10 μL. Chromatograms were acquired at 265, 280, 330, and 360 nm by the DAD detector. Mass spectra were measured between *m*/*z* 100 and 1000 in positive ionization mode (ESI^+^) at a scan rate of 1.06 s/cycle and monitored by a diode array detector. The mass spectrometric conditions were as follows: capillary voltage = 4000 V; drying gas flow = 10 L/min (N_2_); nebulizer pressure = 30 psig; drying gas temperature = 350 °C.

### 3.5. Analysis of Floral Scent Using HP-SPME-GC-MS

A 50/30 μm divinylbenzene/carboxen/polydimethylsiloxane coated SPME fiber (Supelco Inc., Bellefonte, PA, USA) attached to a multipurpose SPME autosampler (MPS 2, Gerstel, Mühlheim, Germany) was used to extract floral volatiles. The SPME fiber was conditioned at 250 °C for 5 min before use and also between the analyses. A sample (1 g) was placed in a headspace vial (22.5 × 75 mm, PTFE/silicone septum, screwed aluminum cap). The tightly capped vial was stirred for 20 min in a thermostatic bath (50 °C) and fibers were exposed to the headspace of the vial for 20 min. Then SPME fibers were injected into the port of a GC-MS system (Agilent GC 6890, Agilent Technologies Inc., Palo Alto, CA, USA) coupled to a mass-selective detector (Agilent GC 6890), and retained for thermal desorption at 250 °C for 2 min. A HP-5MS capillary column (30 m × 0.25 mm, 0.25 μm film thickness, Agilent Technologies Co., Santa Clara, CA, USA.) was used with an oven temperature programmed at 40 °C for 1 min, increased to 80 °C at a rate of 20 °C /min, then to 60 °C at 5 °C /min, then to 270 °C at 20 °C /min, and held at 270 °C for 1 min. Helium was used as a carrier gas at a flow rate of 1 mL/min. The MS interface temperature was set at 250 °C, the ion source temperature was 230 °C, the MS quadrupole temperature was 150 °C, and the transfer line temperature was 280 °C. The peaks in the chromatogram were tentatively identified based on comparisons with those in the Wiley 7 NIST 0.5 Library mass spectral search program, version 5.0 (National Institute of Standards and Technology, Gaithersburg, MD, USA).

### 3.6. Evaluation of DPPH Free Radical Scavenging and ABTS Radical Cation Scavenging Activities

The DPPH of each sample was determined by Brand-Williams’s method [15]. Briefly, each sample was suspended in distilled water and 40 μL of the sample was reacted with 160 μL of 0.2 mM DPPH solution. Absorbance measurements were taken 6 min after the reaction at 517 nm using a Benchmark Plus ELISA reader (Bio-Rad, Hercules, CA, USA). The ABTS of each sample was evaluated using the method published by Re et al. [16]. In brief, the ABTS was measured by pre-formed radical monocation. The mixtures, along with 7.4 mM ABTS solution and 2.6 mM potassium persulfate, were incubated at room temperature in the dark for 24 h. The ABTS solution was diluted with phosphate-buffered saline (pH 7.4) to achieve an absorbance of 0.7 ± 0.02 at 734 nm. Each sample was suspended in distilled water and 40 μL of the sample was reacted with 160 μL of the ABTS solution. Absorbance was taken 6 min after the reaction at 734 nm using the Benchmark Plus ELISA reader (Bio-Rad, Hercules, CA, USA).

### 3.7. Statistical Aalysis

Each experiment was done in triplicate and all data are presented as the mean ± standard deviation (SD). Multiple comparisons were carried out using Duncan’s multiple range tests for the content of 11 standard compounds. The significant level was set at *p* < 0.05.

## 4. Conclusions

In the present study, bioactive compounds such as flavonoids, phenolic acids, and anthocyanins were profiled and quantified in two different color chrysanthemum flowers. As a result of comparative research between them, the infusion of ADC, which had dark purple colored petals, was a prominent source of anthocyanins because of its high anthocyanin content, which were not detected in the commercial yellow chrysanthemum cultivar GG flower tea. In contrast, the GG infusion was considered a good source of flavonoids owing to a very high content of luteolin-7-*O*-β-glucoside. The differences in the extraction of bioactive compounds by infusing time and temperature showed that GG tea was not significantly affected by them, but ADC tea was much affected by infusing time. As the fragrance ingredient is also important for the flower tea, their volatile compounds were analyzed and compared, and their distribution and content were found to be different. Although differences between the teas were detected, they both showed strong ABTS radical scavenging activity and moderate DPPH radical scavenging activity (ADC tea had slightly higher activity than GG tea). Taken together, both ADC and GG are good breeding materials for high quality tea cultivars. Furthermore, as a new tea cultivar with potent antioxidants, anthocyanin and a distinctive color and aroma, the worthy of breeding of ADC can be enhanced. The present study may be a reference for understanding the differences in bioactive compounds and floral scents for purple and yellow chrysanthemum flowers, and for exploring a method of identifying and evaluating flower tea cultivars.

## Figures and Tables

**Table 1:** Flavonoids and phenolic acids identified from the infusions of two different color chrysanthemum flowers by HPLC-DAD/ESIMS methods.

Peak	*t*_R_ (min)	λ_max_ (nm)	[M + H]^+^ (*m*/*z*)	MS^n^ (*m*/*z*)	Identification
1	8.4	275, 350	449	nd ^3^	Luteolin-glucoside ^1^
2	9.9	220, 325	355	163	Chlorogenic acid ^2^
3	23.7	285, 350	465	nd ^3^	Quercetin-glucoside ^1^
4	25.1	270, 350	449	nd ^3^	Luteolin-7-*O*-β-glucoside ^2^
5	25.8	265, 350	463	nd ^3^	Luteolin-glucuronide ^1^
6	28.6	225, 325	517	499/163	Dicaffeoylquinic acid ^1^
7	29.4	265, 350	551	295	unknown
8	31.5	220, 325	517	499/163	3,5-Dicaffeoylquinic acid ^2^
9	32.2	265, 340	433	nd ^3^	Apigenin-7-*O*-β-glucoside ^2^
10	33.7	255, 350	535	nd ^3^	Luteolin-malonylglucoside ^1^
11	34.6	225, 325	517	499/163	Dicaffeoylquinic acid ^1^
12	35.8	265, 350	477	nd ^3^	Diosmetin-glucuronide ^1^
13	41.6	265, 335	519	nd ^3^	Apigenin-malonylglucoside ^1^
14	43.3	250, 350	549	nd ^3^	Diosmetin-malonyglucoside ^1^
15	44.8	265, 330	593	nd ^3^	Linarin ^2^
16	50.8	265, 330	447	nd ^3^	Acacetin-7-*O*-β-glucoside ^2^
17	52.3	265, 350	287	nd ^3^	Luteolin ^2^
18	59.3	270, 335	533	nd ^3^	Acacetin-malonylglucoside ^1^
19	64.6	250, 260, 350	271	nd ^3^	Apigenin ^2^

^1^ Provisionally identified from UV/Vis and mass spectra data. ^2^ Identification was confirmed by comparison with standards. ^3^ Not detected.

**Table 2:** Anthocyanins identified from purple color chrysanthemum flower (ADC) infusions by HPLC-DAD/ESIMS methods.

Peak	*t*_R_ (min)	λ_max_ (nm)	[M]^+^ (*m*/*z*)	MS^n^ (*m*/*z*)	Identification
20	11.9	520	449	nd ^1^	Cyanidin-3-*O*-glucoside
21	14.9	520	535	nd ^1^	Cyanidin 3-*O*-(6″-malonylglucoside)
22	16.8	520	287	nd ^1^	Cyanidin

^1^ Not detected.

**Table 3:** Calibrations and detection limits for 11 standard compounds.

Comps	Calibration Curve ^1^	Correlation Coefficient (*r*^2^)	Linear Range (μg/mL)	LOD ^2^ (μg/mL)	LOQ ^3^ (μg/mL)
**2**	*y* = 18.675*x* − 17.907	0.9994	25–100	0.178	0.539
**4**	*y* =14.105*x* − 16.180	0.9993	25–100	0.267	0.811
**8**	*y* = 22.766*x* − 25.32	0.9991	25–100	0.601	1.822
**9**	*y* = 33.317*x* − 9.170	0.9992	25–100	0.542	1.644
**15**	*y* = 21.849*x* − 21.340	0.9994	25–100	0.716	2.168
**16**	*y* = 14.277*x* + 20.120	0.9991	25–100	0.206	0.624
**17**	*y* = 23.094*x* + 5.220	0.9995	25–100	0.244	0.738
**19**	*y* = 36.953*x* − 46.980	0.9992	25–100	0.575	1.742
**20**	*y* = 44.087*x* − 46.980	0.9963	10–250	0.374	1.134
**21**	*y* = 32.221*x* − 120.13	0.9993	10–250	0.307	0.931
**22**	*y* = 44.603*x* − 381.8	0.9957	10–250	0.444	1.345

^1^*y* and *x* are peak area and concentration (μg/mL), respectively. ^2^ LOD was defined as the concentration that could be detected at a signal-to-noise ratio of 3. ^3^ LOQ was defined as the concentration that could be detected at a signal-to-noise ratio of 10.

**Table 4:** Content (mg/g) of standard compounds in the infusions of two different color chrysanthemum flowers.

Compounds	Infusion Condition							
	ADC ^1^ 75 °C 2 min	ADC 75 °C 4 min	ADC 100 °C 2 min	ADC 100 °C 4 min	GG ^1^ 75 °C 2 min	GG 75 °C 4 min	GG 100 °C 2 min	GG 100 °C 4 min
**2**	4.073 ± 0.540bc ^4^	4.678 ± 0.699b	4.221 ± 0.573b	8.034 ± 1.374a	3.050 ± 0.786c	3.587 ± 0.292bc	3.094 ± 0.915c	3.795 ± 0.551bc
**4**	4.478 ± 0.586d	5.617 ± 0.518d	5.458 ± 1.003d	11.867 ± 1.778c	15.627 ± 3.786b	18.206 ± 0.923ab	16.134 ± 4.219b	20.827 ± 5.393a
**8**	4.900 ± 1.508d	5.178 ± 0.558cd	5.260 ± 0.666cd	9.561 ± 2.078a	7.279 ± 1.724b	8.110 ± 0.530ab	7.058 ± 2.113bc	8.640 ± 2.015ab
**9**	0.713 ± 0.137d	0.979 ± 0.134d	0.978 ± 0.192d	2.106 ± 0.402c	4.319 ± 0.980b	5.125 ± 0.198ab	4.585 ± 1.318b	5.961 ± 1.243a
**15**	0.630 ± 0.327b	0.668 ± 0.104b	0.732 ± 0.138b	1.294 ± 0.219a	nd ^3^ c	nd ^3^ c	nd ^3^ c	nd ^3^ c
**16**	1.252 ± 0.202c	1.847 ± 0.302b	2.176 ± 0.753b	5.077 ± 0.815a	1.130 ± 0.196c	1.082 ± 0.366c	1.263 ± 0.160c	0.964 ± 0.323c
**17**	1.146 ± 0.353b	1.146 ± 0.185b	0.885 ± 0.550b	1.747 ± 0.466a	0.253 ± 0.137c	0.341 ± 0.215c	0.535 ± 0.196c	0.478 ± 0.288c
**19**	nd ^3^ b	nd ^3^ b	nd ^3^ b	nd ^3^ b	0.400 ± 0.135a	0.403 ± 0.081a	0.360 ± 0.079a	0.346 ± 0.038a
**20**	0.531 ± 0.075b	0.763 ± 0.110b	0.673 ± 0.247b	1.975 ± 0.216a	nd ^3^ c	nd ^3^ c	nd ^3^ c	nd ^3^ c
**21**	0.947 ± 0.173c	3.680 ± 0.640b	2.623 ± 0.934bc	8.850 ± 2.114a	nd ^3^ d	nd ^3^ d	nd ^3^ d	nd ^3^ d
**22**	0.535 ± 0.103c	1.151 ± 0.158b	0.710 ± 0.251c	2.054 ± 0.178a	nd ^3^ d	nd ^3^ d	nd ^3^ d	nd ^3^ d
T_PA_^2^	8.973 ± 2.048	9.856 ± 1.258	9.480 ± 1.238	17.595 ± 3.453	10.328 ± 2.510	11.696 ± 0.822	10.152 ± 3.029	12.435 ± 2.566
T_F_^2^	7.919 ± 1.605	10.257 ± 1.245	10.229 ± 2.636	22.090 ± 3.680	21.728 ± 5.234	25.157 ± 1.783	22.877 ± 5.972	28.576 ± 7.286
T_A_^2^	1.994 ± 0.351	5.594 ± 0.909	4.006 ± 1.433	12.879 ± 2.508	nd ^3^	Nd ^3^	nd ^3^	nd ^3^
T_T_^2^	18.185 ± 4.004	25.707 ± 3.411	23.716 ± 5.307	52.564 ± 9.641	32.057 ± 7.744	36.853 ± 2.605	33.029 ± 9.001	41.011 ± 9.852

^1^ ADC and GG represent the dark purple chrysanthemum cultivar (ARTI-Dark Chocolate) and yellow chrysanthemum cultivar (Gamguk), respectively. ^2^ T_PA_, T_F_, T_A_, and T_T_ represent the sum quantities of phenolic acids, flavonoids, anthocyanins, and total compounds, respectively. ^3^ Not detectable. ^4^ Lowercase letters indicated statistical significance−samples not sharing a letter differed significantly according to Duncan’s multiple range test (*p* < 0.05).

**Table 5:** Antioxidant activities of the infusions of two different color chrysanthemum flowers.

Sample—Infusion Condition (200 μg/mL)	ABTS (% Inhibition)	DPPH (% Inhibition)
ADC—75 °C, 2 min	72.60 ± 0.98	51.10 ± 3.05
ADC—75 °C, 4 min	74.92 ± 7.71	54.92 ± 0.92
ADC—100 °C, 2 min	67.94 ± 7.63	62.07 ± 3.16
ADC—100 °C, 4 min	67.42 ± 1.77	66.20 ± 5.08
GG—75 °C, 2 min	69.91 ± 6.70	30.19 ± 1.39
GG—75 °C, 4 min	61.46 ± 5.04	37.97 ± 2.98
GG—100 °C, 2 min	76.24 ± 4.92	47.18 ± 1.40
GG—100 °C, 4 min	71.53 ± 2.95	43.40 ± 4.81
Ascorbic acid ^1^	94.56 ± 0.12	58.99 ± 0.42

^1^ Ascorbic acid was used as a positive control.

**Table 6:** Identification of floral scent volatile compounds in two different color chrysanthemum flower tea bags.

ADC ^1^					GG ^1^				
*t*_R_ (min)	Identification	Formula	M.W.	% of Total	*t*_R_ (min)	Identification	Formula	M.W.	% of Total
1.50	Lactamide	C_3_H_7_NO_2_	89	1.59	3.38	Hexanal	C_6_H_12_O	100	0.14
2.50	Pentanal	C_5_H_10_O	89	0.26	3.76	1,6-Dimethylhepta-1,3,5-triene	C_9_H_14_	122	0.04
3.29	1-Octene	C_8_H_16_	112	0.75	4.93	β-Thujene	C_10_H_16_	136	0.14
3.38	Hexanal	C_6_H_12_O	100	0.64	5.05	α-Pinene	C_10_H_16_	136	0.44
3.97	2,6-Dimethyl-3,5-heptadien-2-ol	C_9_H_16_O	140	0.11	5.74	1-Octen-3-ol	C_8_H_16_O	128	1.27
4.25	2-Methyl-acetate 1-butanol	C_7_H_14_O_2_	130	0.17	5.83	Sulcatone	C_8_H_14_O	126	0.49
4.63	Santolina triene	C_10_H_16_	136	0.21	5.92	β-Myrcene	C_10_H_16_	136	0.93
4.89	Tricyclene	C_10_H_16_	136	0.12	6.00	1-Ethyl-3-methyl-benzene	C_9_H_12_	120	0.47
4.94	β-Thujene	C_10_H_16_	136	1.07	6.19	α-Phellandrene	C_10_H_16_	136	0.42
5.06	α-Pinene	C_10_H_16_	136	4.29	6.55	o-Cymene	C_10_H_14_	134	0.99
5.29	Camphene	C_10_H_16_	136	3.11	6.72	Eucalyptol	C_10_H_18_O	154	3.93
5.66	4(10)-Thujene	C_10_H_16_	136	2.48	7.19	γ-Terpinene	C_10_H_16_	136	0.52
5.73	β-Pinene	C_10_H_16_	136	0.52	7.47	β-Terpineol	C_10_H_18_O	154	1.61
5.82	Sulcatone	C_8_H_14_O	126	0.29	7.81	γ-Pyronene	C_10_H_16_	136	0.25
5.92	β-Myrcene	C_10_H_16_	136	7.44	8.16	γ-Terpinenol	C_10_H_18_O	154	2.47
6.20	α-Phellandrene	C_10_H_16_	136	6.65	8.52	Pseudo-cyclocitral	C_10_H_16_O	152	6.22
6.33	3,4-Dimethyl-styrene	C_10_H_12_	132	0.37	8.71	Chrysanthnone	C_10_H_14_O	150	5.49
6.40	α-Terpinene	C_10_H_16_	136	0.20	9.26	(+)-2-Bornanone	C_10_H_16_O	152	12.28
6.56	o-Cymene	C_10_H_14_	134	4.43	9.71	cis-Verbenol	C_10_H_16_O	152	0.48
6.72	Eucalyptol	C_10_H_18_O	154	16.25	9.88	Terpinen-4-ol	C_10_H_18_O	154	0.94
7.19	γ-Terpinene	C_10_H_16_	136	0.29	10.13	D-Verbenone	C_10_H_14_O	150	0.57
7.37	β-Terpineol	C_10_H_18_O	154	0.39	10.27	α-Terpineol	C_10_H_18_O	154	1.55
7.80	γ-Pyronene	C_10_H_16_	136	0.20	11.31	trans-Verbenyl acetate	C_12_H_18_O_2_	194	16.24
8.08	2-Methyl butyl ester butanoic acid	C_10_H_20_O_2_	172	3.47	12.10	Isopiperitenone	C_10_H_14_O	150	0.44
8.13	Chrysanthenone	C_10_H_14_O	150	1.07	12.41	Bornyl acetate	C_12_H_20_O_2_	196	0.33
8.22	1-Octenyl acetate	C_10_H_18_O_2_	170	0.30	13.25	1,6-Dimethylhepta-1,3,5-triene	C_9_H_14_	122	0.49
8.66	Chrysanthenone	C_10_H_14_O	150	11.34	13.51	2-Methyl-heptyl ester butanoic acid	C_12_H_24_O_2_	200	0.24
9.13	(+)-2-Bornanone	C_10_H_16_O	152	14.33	15.07	β-Elemene	C_15_H_24_	204	7.02
9.54	(−)-Borneol	C_10_H_18_O	154	0.40	15.54	2,3,3-Trimethyl-2-(3-methyl-buta-1,3-dienyl)-cyclohexanone	C_14_H_22_O	206	1.02
					15.63	2-Pinen-4-one	C_10_H_14_O	150	0.26
					15.73	Caryophyllene	C_15_H_24_	204	0.36
					16.50	cis-β-Farnesene	C_15_H_24_	204	0.78
					16.69	Limonen-6-ol, pivalate	C_15_H_24_O_2_	236	0.14
					16.81	4-(2,2-Dimethyl-6-methylenecyclohexylidene)-3-methylbutan-2-one	C_14_H_22_O	206	0.28
					17.02	4,11-Selinadiene	C_15_H_24_	204	0.13
					17.18	α-Curcumene	C_15_H_22_	202	1.37
					17.33	β-Selinene	C_15_H_24_	204	1.04
					17.53	α-Selinene	C_15_H_24_	204	0.61
					17.76	β-Bisabolene	C_15_H_24_	204	0.27
					18.12	β-Sesquiphellandrene	C_15_H_24_	204	0.31
					18.83	Isoaromadendrene epoxide	C_15_H_24_O	220	0.12
					19.58	Caryophyllene oxide	C_15_H_24_O	220	0.20
					19.87	Longifolenaldehyde	C_15_H_24_O	220	0.58
					21.31	Longipinocarvone	C_15_H_22_O	218	0.20
					21.62	Ledene oxide-(II)	C_15_H_24_O	220	0.32
					21.97	trans-Longipinocarveol	C_15_H_24_O	220	0.18
					23.71	Aromadendrene oxide-(2)	C_15_H_24_O	220	0.17
					23.84	Isoaromadendrene epoxide	C_15_H_24_O	220	0.14
					25.44	cis-*Z*-α-Bisabolene epoxide	C_15_H_24_O	220	0.05

^1^ ADC and GG represent the dark purple chrysanthemum cultivar (ARTI-Dark Chocolate) and yellow chrysanthemum cultivar (Gamguk), respectively.
